# Integrated mPD‐L1 and metabolic analysis identifies new prognostic subgroups in lung cancers with wild‐type EGFR

**DOI:** 10.1002/ctm2.612

**Published:** 2021-12-19

**Authors:** Guosheng Wang, Weilei Hu, Yundi Chen, Yuan Wan, Qiang Li

**Affiliations:** ^1^ Department of Pulmonary and Critical Care Medicine, Shanghai East Hospital, School of Medicine Tongji University Shanghai China; ^2^ The Pq Laboratory of Micro/Nano BiomeDx, Department of Biomedical Engineering Binghamton University—SUNY Binghamton New York USA; ^3^ Institute of Translational Medicine Zhejiang University Hangzhou China

Dear Editor,

Metabolic reprogramming, especially changes in glycolysis and cholesterogenesis pathways, has been reported to affect tumour prognosis.^1^ Interestingly, there is an intricate relationship between metabolic changes and immune checkpoints in the tumour microenvironment (TME),^2^ but their interaction and effects on the prognosis of lung cancer patients remain poorly understood. In this work, we established a novel stratification framework based on combined analysis of PD‐L1 mRNA (mPD‐L1) expression and glycolysis/cholesterol metabolic signatures, which stratified epidermal growth factor receptor (EGFR) wild‐type lung cancers into metabolic subtypes with significantly different prognoses. We also created a visualization website called glycolysis/cholesterol metabolism axis and PD‐L1 mRNA expression (GCP) (https://www.liqlab.cn/gcp) for this stratification approach.

We first investigated the impact of the three metabolic subtypes on prognosis at different PD‐L1 expression levels. In the mPD‐L1^low^ group, cholesterogenic cases had a significantly worse OS and progression free survival (FPS) (mOS: 2.9 years; mFPS: 3.7 years) compared to glycolytic (mOS and mPFS were not reached) and quiescent cases (mOS: 5.5 years, mPFS was not reached). However, in the mPD‐L1^high^ group, cases belonging to the glycolytic subtype had a significantly worse OS and FPS (mOS: 3.3 years; mFPS: 2.3 years) than the cholesterogenic and quiescent subtypes (mOS: 7.1 years, mPFS: 7.2 years). Of note, no difference was observed among metabolic subtypes for the mPD‐L1^med^ group (Figure 1C and Table S4). Furthermore, for the cholesterogenic subtype, higher PD‐L1 levels were associated with a better prognosis, whereas opposite findings were observed for the glycolytic subtype (Figure 1D).

**FIGURE 1:**
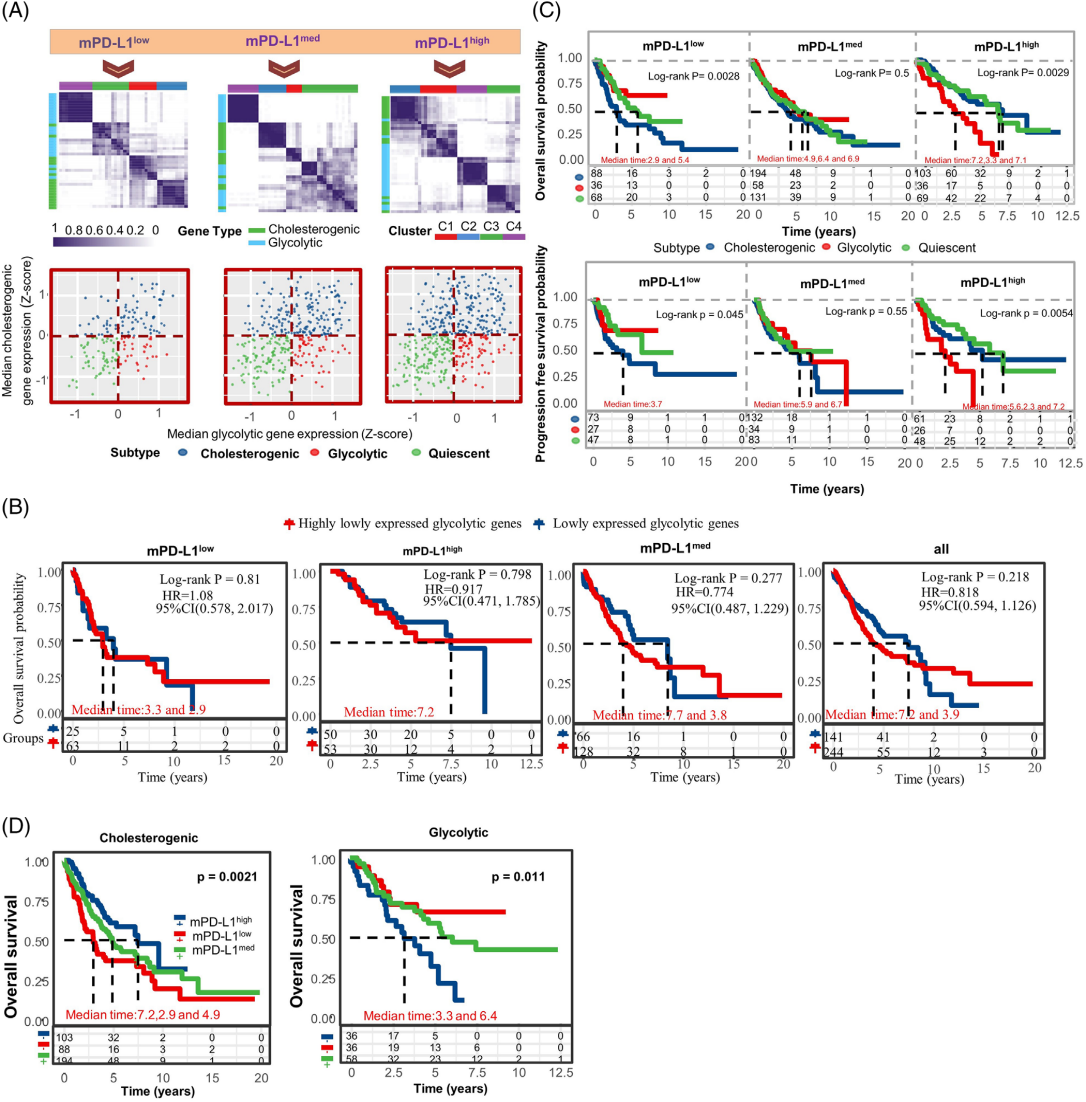
Clinical prognostic of EGFR wild type lung cancers with different metabolic subtypes. (A) Stratification of mPD‐L1^low^, mPD‐L1^med^, and mPD‐L1^high^ groups based on gene expression of glycolysis/cholesterol synthesis axis. Heatmap (upper) showing results of consensus clustering analysis for genes involved in glycolytic and cholesterogenic processes in mPD‐L1^low^ (*k* = 4, *n* = 192), mPD‐L1^med^ (*k* = 4, *n* = 383), and mPD‐L1^high^ (*k* = 4, *n* = 208) groups of EGFR wild‐type lung cancers. Scatter plot (down) illustrating median expression level of co‐expressed genes associated with glycolytic (x‐axis) and cholesterogenic (y‐axis) processes for all samples. The expression of these genes was used to establish metabolic subtypes. (B) Overall survival for groups with highly expressed cholesterogenic genes and high or low glycolytic gene expression. (C) Kaplan–Meier survival analysis in mPD‐L1^low^, mPD‐L1^med^, or mPD‐L1^high^ groups stratified by metabolic subtype. Upper panel, overall survival (OS) analysis; lower panel, progression free survival (PFS) analysis; Log‐rank test *p* values are shown. (D) Overall survival for glycolytic and cholesterogenic groups with different mPD‐L1 expression

For the mPD‐L1^high^ group, univariate cox regression analysis revealed that glycolytic subtype, pT stage and pTNM stage were correlated with OS and PFS. During multivariate Cox regression analysis, glycolytic subtype (hazard ratio [HR], 2.62; 95% confidence interval [CI], 1.19–5.73; *p* = 0.02) and pT stage (HR, 2.69; 95% CI, 1.08–6.73; *p* = 0.03) were found to be independent predictors of OS after adjusting for typical clinicopathologic factors (Table 1). For the mPD‐L1^low^ group, univariate cox regression analysis uncovered that age, gender, smoking, glycolytic subtype and cholesterogenic subtype were correlated with OS, and only cholesterogenic subtype was related to PFS. Multivariate analysis using the Cox regression model further demonstrated that only the cholesterogenic subtype was independently prognostic for OS (HR, 2.07; 95% CI, 1.13–3.78; *p* = 0.02) (Table 1). Overall, the above findings suggest that in EGFR wild‐type non‐small cell lung cancer (NSCLC), different metabolic subtypes have distinct prognostic outcomes based on PD‐L1 expression levels. A more aggressive phenotype could be associated with predominantly cholesterogenic tumours than those with predominantly glycolytic phenotype in EGFR wild‐type NSCLC poorly expressing PD‐L1.

**TABLE 1:** Univariate and multivariate regression analysis of different clinical parameters and metabolic subtypes

PD‐L1^low^		OS					PFS			
Variable	Univariate, *p*	Multivariate (HR, 95% CI)			*p*‐value	Univariate, *p*	Multivariate (HR, 95% CI)			*p*‐value
Age (>60 vs. ≤60)	*0.026*	1.003	0.971	1.036	0.866	0.52	1.004	0.97	1.04	0.812
Gender (male vs. female)	*0.015*	1.668	0.937	2.969	0.082	0.16	1.834	0.977	3.443	0.059
pT_stage (T3/T4 vs. T1/T2)	0.73	1.135	0.523	2.46	0.749	0.646	2.049	0.85	4.935	0.11
pN_stage (N1/N2/N3 vs. N0)	0.569	0.776	0.425	1.418	0.409	0.184	0.577	0.291	1.143	0.115
pM_stage (M1/MX vs. M0)	0.223	1.719	0.791	3.735	0.171	0.402	1.419	0.601	3.35	0.425
pTNM_stage (III/IV vs. I/II)	0.597	1.291	0.637	2.616	0.479	0.072	1.514	0.664	3.451	0.324
Smoking (Yes vs. No)	*0.002*	1.951	0.739	5.151	0.177	0.22	2.012	0.598	6.769	0.259
Histology (LUAD vs. LUSC)	0.108	1.04	0.618	1.747	0.883	0.192	1.144	0.659	1.985	0.633
Glycolytic (glycolytic vs. quiescent)	*0.029*	0.723	0.326	1.602	0.424	0.351	0.707	0.292	1.715	0.444
Cholesterogenic (cholesterogenic vs. quiescent)	*0.001*	*2.065*	*1.129*	*3.779*	*0.019*	*0.015*	1.84	0.973	3.482	0.061

**PD‐L1^high^**		**OS**					**PFS**			
Variable	Univariate, *p*	Multivariate (HR, 95% CI)			*p*‐value	Univariate, *p*	Multivariate (HR, 95% CI)			*p*‐value
Age (>60 vs. ≤60)	0.15	1.009	0.98	1.038	0.555	0.536	0.995	0.959	1.033	0.8
Gender (male vs. female)	0.853	0.969	0.491	1.909	0.926	0.324	0.919	0.446	1.894	0.818
pT_stage (T3/T4 vs. T1/T2)	*<0.0001*	*2.694*	*1.079*	*6.725*	*0.034*	*0.001*	2.256	0.88	5.782	0.09
pN_stage (N1/N2/N3 vs. N0)	0.193	1.062	0.531	2.126	0.864	0.098	0.828	0.402	1.705	0.609
pM_stage (M1/MX vs. M0)	0.761	1.428	0.522	3.902	0.488	0.773	0.895	0.275	2.911	0.853
pTNM_stage (III/IV vs. I/II)	*<0.0001*	2.245	0.851	5.922	0.102	*0.008*	2.151	0.794	5.824	0.132
Smoking (yes vs. no)	0.153	0.437	0.163	1.176	0.101	0.32	*0.171*	*0.051*	*0.574*	*0.004*
Histology (LUAD vs. LUSC)	0.153	0.827	0.419	1.631	0.584	0.951	1.27	0.612	2.635	0.521
Glycolytic (glycolytic vs. quiescent)	*0.001*	*2.616*	*1.194*	*5.732*	*0.016*	*0.002*	1.935	0.861	4.347	0.11
Cholesterogenic (cholesterogenic vs. quiescent)	0.181	0.977	0.507	1.882	0.944	0.603	0.877	0.414	1.856	0.731

*Note*: Significant *p* < 0.05 is given in italic.

Abbreviations: CI, confidence interval; HR, hazard ratio; OS, overall survival; PFS, progression free survival.

We subsequently focused on the mPD‐L1^low^ group of EGFR wild‐type lung cancers. Analysis of clinical characteristics showed that cholesterogenic cases were more likely to have a smoking history and higher T and N scores (Figure S3A). For immune profiles, significantly lower immune scores and lower tumour infiltration of endothelial cells, macrophages and B cells were found in the cholesterogenic subtype than in the quiescent subtype (Figure S3B,C). Higher expression levels of CD274 and TIGIT were observed in cholesterogenic cases than glycolytic and quiescent cases (Figure S3D). We previously found hypermethylation and low expression of multiple tumour suppressor genes (TSGs) in EGFR wild‐type NSCLC poorly expressing PD‐L1.^3^ In the present study, the expression of TSGs ADAMTS8 (Adam metallopeptidase with thrombospondin type 1 motif 8), CDO1 (cysteine dioxygenase type 1), and GATA5 (GATA binding protein 5) varied across metabolic subtypes. This finding suggested possible heterogeneity in carcinogenic mechanisms across metabolic subtypes (Figure S3E).

Interestingly, mutation analysis revealed that the mutation frequency and types varied significantly across the metabolic subtypes. Multiple genes likely involved included TP53, TTN, ZFHX4, ROS1, DNAH9, PCDH15, ALK and KRAS (Figure 2A–C). Next, cancer hallmarks analysis showed that tumour proliferation signature, G2M checkpoint hallmark, MYC targets hallmark and mRNAsi signatures were more active in cholesterogenic cases than glycolytic cases (Figure 2D). The association between different cancer hallmarks further suggested that each metabolic subtype had a unique tumourigenic development pattern. (Figure 2E). We also explored the expression of some therapeutic targets. We noticed that the mitochondrial pyruvate carrier 1 (MPC 1) gene expression level was profoundly lower in the glycolytic subtype versus the cholesterogenic subtype (Figure 2F).^4^ Recently, methylenetetrahydrofolate dehydrogenase 2 (MTHFD2) was found to promote PD‐L1‐mediated tumour immune resistance. Proprotein convertase subtilisin/keying type 9 (PCSK9) has been documented to block intratumoural infiltration by T cells.^5^, ^6^ In the present study, we found that cholesterogenic cases expressed significantly higher levels of MTHFD2 and PCSK9 than other cases (Figure 2G). Furthermore, as shown in Figure 2H, 10 pathways were significantly enriched in the cholesterogenic group (false discovery rate (FDR) < 0.05).

**FIGURE 2:**
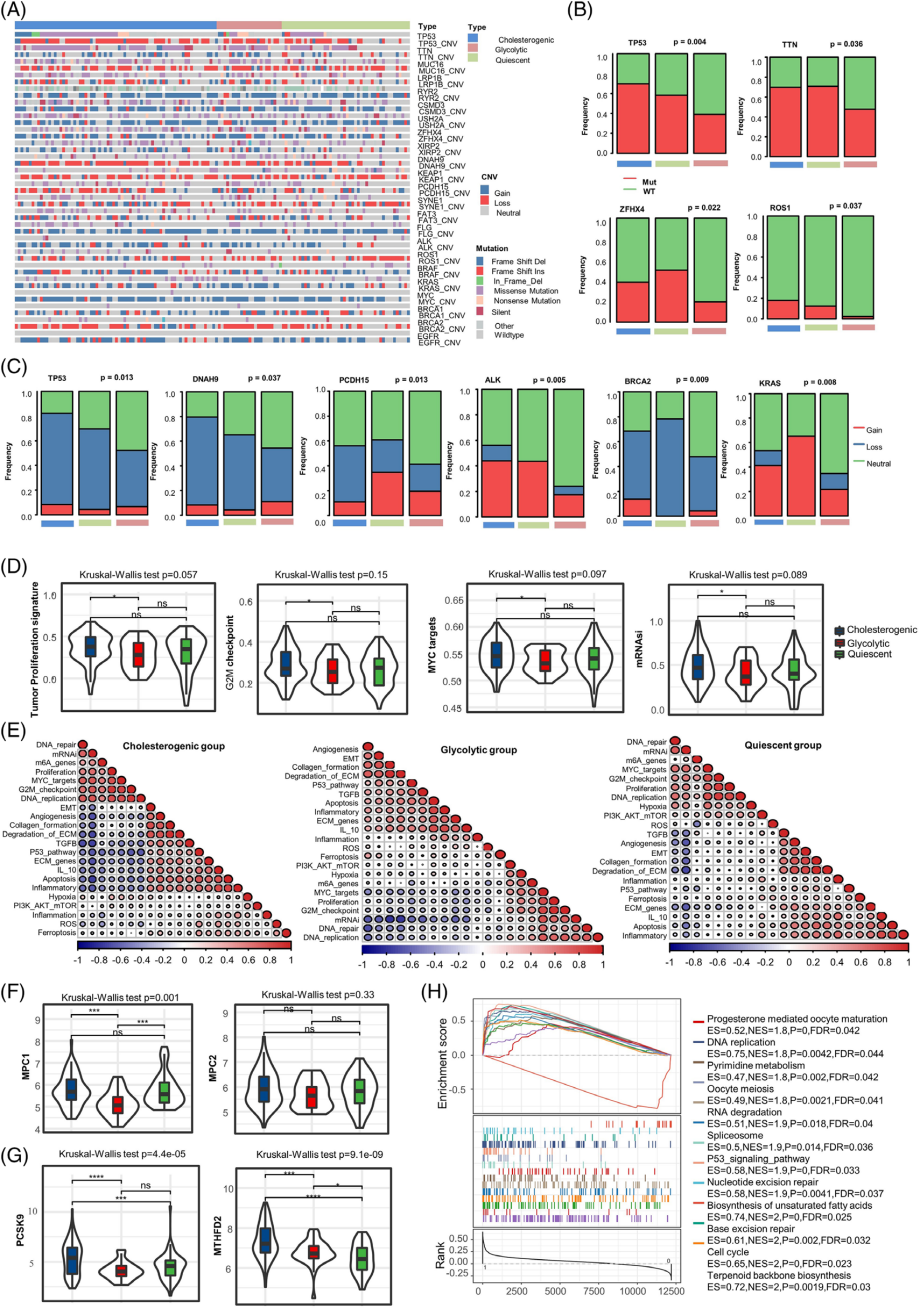
Mutational landscape and cancer hallmarks across the metabolic subtypes of mPD‐L1^low^ group of EGFR wild type NSCLC. (A) Oncoprint illustrating the distribution of somatic mutation (single nucleotide variation/indel) and copy number variation (CNV) events influencing frequently mutated genes in NSCLC across the metabolic subtypes. (B) The distribution of genes with somatic mutations across the metabolic subtypes. (C) The distribution of genes with copy number variations across the metabolic subtypes. (D) The different scores of cancer hallmarks in metabolic subtypes. (E) The association between cancer hallmarks in each metabolic subtype. (F) The expression levels of MPC1 and MPC2 in all metabolic subtypes. (G) The expression levels of MTHFD2 and PCSK9 in all metabolic subtypes. (H) Significantly enriched gene sets in the cholesterogenic group (FDR < 0.05). Kruskal–Wallis test was performed to compare the four subgroups. Wilcoxon test was used to compare two paired groups. (*p < 0.05, *p < 0.01, ***p < 0.001 and ****p < 0.0001)

We then evaluated the stratification framework in other cancer types. Network topology analysis was used to identify co‐expressed pathway‐specific genes in 12 cancer types (Figure 3A). In mPD‐L1^high^ groups of other cancer types, significant differences in survival were observed for bladder cancer patients (log‐rank *p *= 0.024) as glycolytic cases exhibited favorable OS (mOS was not reached). In mPD‐L1^low^ groups, significant differences in survival across the metabolic subtypes were observed in Kidney renal clear cell carcinoma (kidney renal clear cell carcinoma (KIRC) log‐rank *p *= 0.0013) and thyroid carcinoma (THCA) patients (log‐rank *p *= 0.0095). For KIRC, cholesterogenic cases had better OS (mOS was not reached), whereas glycolytic (mOS: 3.5 years) and quiescent cases (mOS: 4.1 years) had worse OS (mOS: 2 years). For THCA, cholesterogenic and quiescent cases had better OS (mOS was not reached), and glycolytic cases (mOS was not reached) had relatively worse OS (Figure 3A–C). Interestingly, the cholesterogenic subtype was an independent clinical factor only in KIRC (Table S5). Altogether demonstrated that despite the distinct genomic signatures and TME factors specific to each cancer type, tumour metabolic dependencies varied with PD‐L1 expression levels.

**FIGURE 3:**
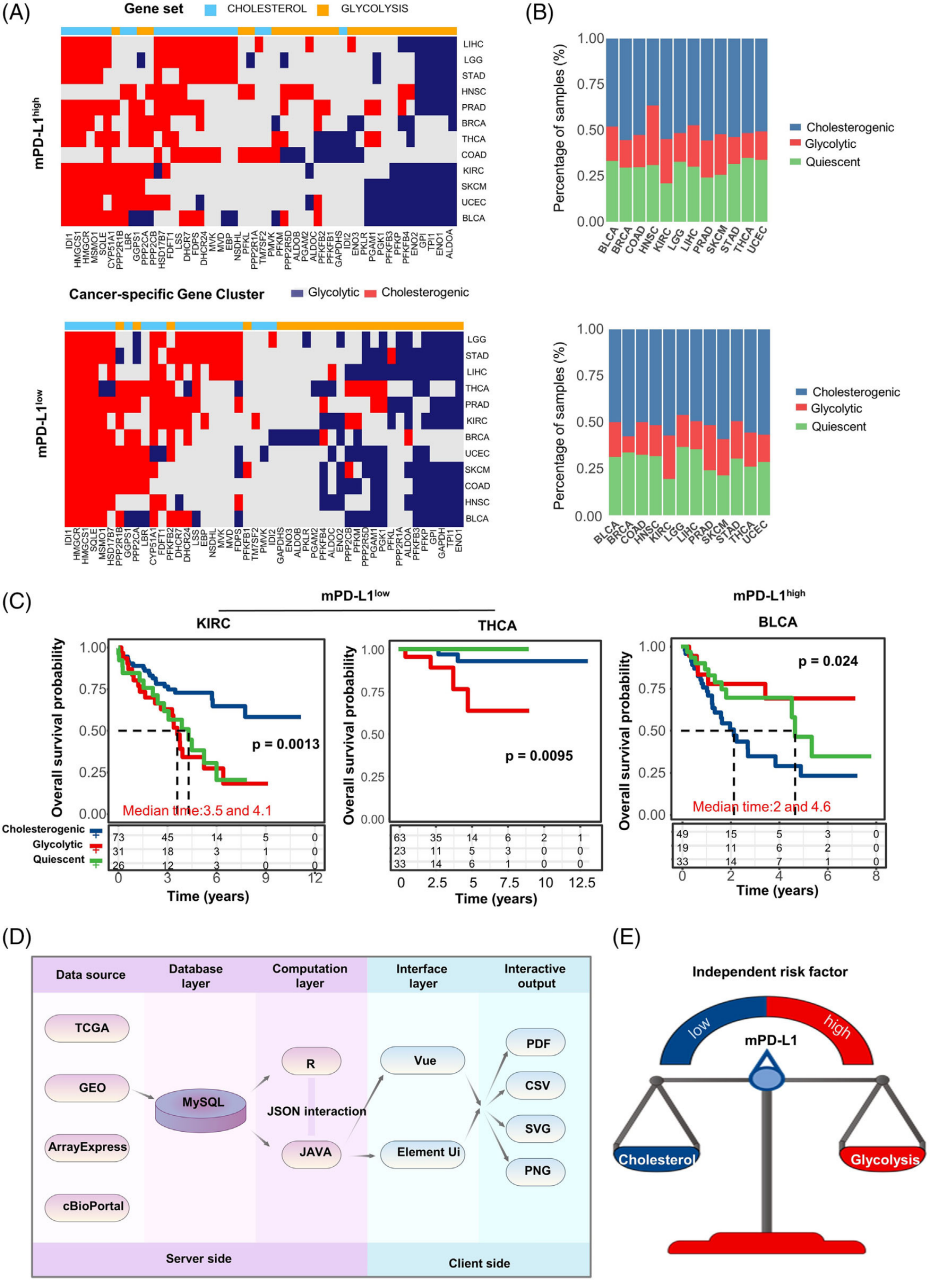
The effectiveness of stratification framework in pan‐cancer. (A) Heatmap displaying the results of consensus clustering analysis for genes involved in glycolysis and cholesterogenic processes for each cancer type. (B) Bar plots illustrating the proportion of metabolic subtypes in different cancer types. (C) Kaplan–Meier survival analysis curves displaying variations in median OS between metabolic subgroups in KIRC with low expression of mPD‐L1, THCA with low expression of mPD‐L1, and bladder urothelial carcinoma with high expression of mPD‐L1. (D) Design and workflow of interactive online webtool named GCP. (E) The interaction patterns of PD‐L1 mRNA and altered glycolysis/cholesterol metabolism axis ultimately affect patient prognosis

To make the stratification framework more convenient and user‐friendly, we also created an online tool called GCP (https://www.liqlab.cn/gcp), where investigators can submit transcriptome data of their own samples and obtain the stratification results with a single click (Figure 3D and Figure S4).

In summary, our stratification framework indicates the role of metabolic phenotype in the prognosis of EGFR wild‐type lung cancer, which is also related to the expression of PD‐L1 (Figure 3E). Moreover, this can help improve the current management of EGFR wild‐type lung cancer patients. Meanwhile, it also provided clues for the selection of candidate drugs for combination treatment strategy using PD‐1/PD‐L1 inhibitors in these two subgroups of EGFR wild‐type patients (mPD‐L1^low^/cholesterogenic; mPD‐L1^high^/ glycolytic).

## CONFLICT OF INTEREST

The authors declare no conflict of interest.

## Supporting information



SUPPORTING INFORMATION

SUPPORTING INFORMATION

SUPPORTING INFORMATION

SUPPORTING INFORMATION

SUPPORTING INFORMATION

## References

[1] Karasinska JM , Topham JT , Kalloger SE , et al. Altered gene expression along the glycolysis‐cholesterol synthesis axis is associated with outcome in pancreatic cancer. Clin Cancer Res. 2020;26(1):135‐146. 31481506 10.1158/1078-0432.CCR-19-1543

[2] Li X , Wenes M , Romero P , Huang SC‐C , Fendt S‐M , Ho P‐C . Navigating metabolic pathways to enhance antitumour immunity and immunotherapy. Nat Rev Clin Oncol. 2019;16(7):425‐441. 30914826 10.1038/s41571-019-0203-7

[3] Hu W , Wang G , Yarmus LB , Wan Y . Combined methylome and transcriptome analyses reveals potential therapeutic targets for EGFR wild type lung cancers with low PD‐L1 expression. Cancers. 2020;12(9):2496. 10.3390/cancers12092496 PMC7563876 32899191

[4] Schell JC , Olson KA , Jiang L , et al. A role for the mitochondrial pyruvate carrier as a repressor of the Warburg effect and colon cancer cell growth. Mol Cell. 2014;56(3):400‐413. 25458841 10.1016/j.molcel.2014.09.026 PMC4268416

[5] Liu X , Bao X , Hu M , et al. Inhibition of PCSK9 potentiates immune checkpoint therapy for cancer. Nature. 2020;588(7839):693‐698. 33177715 10.1038/s41586-020-2911-7 PMC7770056

[6] Shang M , Yang H , Yang R , et al. The folate cycle enzyme MTHFD2 induces cancer immune evasion through PD‐L1 up‐regulation. Nat Commun. 2021;12(1):1940. 33782411 10.1038/s41467-021-22173-5 PMC8007798

