# Supplementary material for: Integrated mPD‐L1 and metabolic analysis identifies new prognostic subgroups in lung cancers with wild‐type EGFR
Source: Clin Transl Med. 2021 Dec 19;11(12):e612. doi: 10.1002/ctm2.612 (PMC8684767; doi:10.1002/ctm2.612)
Supplement: Supplementary file 1 — SUPPORTING INFORMATION [file CTM2-11-e612-s001.docx]

**Tables**

**Table S1. Overview of data sources under study.**

| Data Sources | Number of  Patients | Platform | Data Type | Number of  Patients | |
| --- | --- | --- | --- | --- | --- |
| TCGA-LUAD | 522 | — | RNAseq | | 197 |
| TCGA-LUSC | 504 | — | RNAseq | | 278 |
| GSE13213 | 117 | GPL6480 | Agilent-014850 Whole Human Genome Microarray 4x44K G4112F (Probe Name version) | | 72 |
| GSE29016 | 72 | GPL6947 | Illumina HumanHT-12 V3.0 expression beadchip | | 64 |
| GSE31210 | 226 | GPL570 | [HG-U133_Plus_2] Affymetrix Human Genome U133 Plus 2.0 Array | | 68 |
| GSE33072 | 131 | GPL6244 | [HuGene-1_0-st] Affymetrix Human Gene 1.0 ST Array [transcript (gene) version] | | 104 |
| GSE75037 | 83 | GPL6884 | Illumina Human WG-6 v3.0 expression beadchip | | 63 |
| GSE66863 | 121 | GPL14550 | Agilent-028004 SurePrint G3 Human GE 8x60K Microarray (Probe Name Version) | | 102 |
| luad_oncosg_2020 | 305 | — | RNAseq | | 75 |
| E-MTAB-923 | 103 | GPL570 | [HG-U133_Plus_2] Affymetrix Human Genome U133 Plus 2.0 Array | | 42 |

**Table S2. Demographics and clinical characteristics of patients with EGFR wild type lung cancer.**

| Characteristic | | EGFR wild type | | | |
| --- | --- | --- | --- | --- | --- |
|  |  | PD-L1 bottom25% | PD-L1 median 25%~75% | PD-L1 top25% | P-value |
|  |  | (n=192) | (n=383) | (n=208) |  |
| Age (years) | ≤60 | 51 | 106 | 75 | 0.056 |
|  | >60 | 140 | 276 | 132 |  |
| Survival status | Alive | 106 | 213 | 127 | 0.377 |
|  | Dead | 86 | 170 | 81 |  |
| PFS status | NO | 101 | 168 | 86 | 0.645 |
|  | Yes | 46 | 81 | 49 |  |
| Gender | Female | 78 | 174 | 88 | 0.511 |
|  | Male | 114 | 209 | 120 |  |
| Pathologic T | T1 | 40 | 108 | 40 | 0.095 |
|  | T2 | 103 | 162 | 87 |  |
|  | T3 | 17 | 33 | 17 |  |
|  | T4 | 6 | 19 | 3 |  |
| Pathologic N | N0 | 113 | 226 | 103 | 0.541 |
|  | N1 | 32 | 55 | 34 |  |
|  | N2 | 18 | 36 | 10 |  |
|  | N3/NX | 3 | 6 | 1 |  |
| Pathologic M | M0 | 139 | 278 | 127 | 0.629 |
|  | M1/MX | 27 | 42 | 20 |  |
| Clinical staging | StageⅠ | 105 | 229 | 116 | 0.025 |
|  | StageⅡ | 51 | 86 | 67 |  |
|  | StageⅢ | 39 | 58 | 19 |  |
|  | StageⅣ | 5 | 7 | 3 |  |
| Smoking | NO | 31 | 71 | 38 | 0.724 |
|  | Yes | 151 | 287 | 157 |  |

**Table S3. Co-expressed glycolytic and cholesterogenic genes with different expression levels of mPD-L1**

| Coexpressed Glycolytic Genes | | | Coexpressed Cholesterogenic Genes | | |
| --- | --- | --- | --- | --- | --- |
| PD-L1^low^ | PD-L1^med^ | PD-L1^high^ | PD-L1^low^ | PD-L1^med^ | PD-L1^high^ |
| ENO1 | ALDOC | ALDOA | EBP | EBP | EBP |
| ALDOC | ALDOA | ENO1 | NSDHL | FDPS | NSDHL |
| TPI1 | PGK1 | ENO2 | DHCR7 | NSDHL | DHCR7 |
| GPI | PFKFB4 | GAPDH | HMGCR | DHCR7 | FDFT1 |
| PGK1 | TPI1 | GPI | HMGCS1 | HMGCR | HMGCR |
| ENO2 | ENO1 | PFKFB4 | SQLE | HMGCS1 | HMGCS1 |
| PFKFB3 | ENO2 | PFKL | FDFT1 | IDI1 | SQLE |
| ALDOA | GAPDH | PFKP | FDPS | SQLE | MVK |
| GAPDH | GPI | PGK1 | IDI1 | FDFT1 |  |
| PFKFB4 | PFKP | TPI1 | LBR |  |  |
| PFKP |  |  |  |  |  |

**Table S4. Univariate analysis showing the influence of different metabolic types on prognosis at different expression levels of PD-L1**

|  | OS | | | | | |
| --- | --- | --- | --- | --- | --- | --- |
|  | PD-L1^low^ | | PD-L1^med^ | | PD-L1^high^ | |
| Comparison | Log rank p | HR, 95% CI | Log rank p | HR, 95% CI | Log rank p | Hazard ratios |
| Cholesterogenic vs. Glycolytic | 0.0006 | 2.601 (1.311 ~ 5.156) | 0.2641 | 1.294 (0.822 ~ 2.036) | 0.0029 | 0.431 (0.248 ~ 0.750) |
| Glycolytic vs. Quiescent | 0.2677 | 0.664 (0.322 ~ 1.368) | 0.643 | 0.892 (0.552 ~ 1.442) | 0.0035 | 2.461 (1.344 ~ 4.506) |
| Cholesterogenic vs. Quiescent | 0.0155 | 1.783 (1.116 ~ 2.849) | 0.4775 | 1.128 (0.807 ~ 1.577) | 0.9828 | 0.994 (0.594 ~ 1.663) |
|  | PFS | | | | | |
|  | PD-L1^low^ | | PD-L1^med^ | | PD-L1^high^ | |
| Comparison | Log rank p | HR, 95% CI | Log rank p | HR, 95% CI | Log rank p | Hazard ratios |
| Cholesterogenic vs. Glycolytic | 0.1081 | 1.975 (0.861 ~ 4.529) | 0.4002 | 1.322 (0.689 ~ 2.534) | 0.0189 | 0.432 (0.214 ~ 0.870) |
| Glycolytic vs. Quiescent | 0.7627 | 1.157 (0.447 ~ 2.998) | 0.9457 | 0.974 (0.469 ~ 2.025) | 0.0027 | 3.319 (1.513 ~ 7.282) |
| Cholesterogenic vs. Quiescent | 0.0293 | 2.179 (1.081 ~ 4.389) | 0.3097 | 1.296 (0.785 ~ 2.139) | 0.5071 | 1.256 (0.640 ~ 2.466) |

| *Abbrev:* HR, hazard ratio; CI, confidence interval. | |
| --- | --- |
| Significant *p* < 0.05 is highlighted red. |  |

**Table S5. Multivariate regression analysis of different clinical parameters and metabolic types in other cancer types.**

| PD-L1^low^ | KIRC(OS) | | | |
| --- | --- | --- | --- | --- |
| Variable | Multivariate (HR, 95% CI) | | | P-value |
| Age | 1.05 | 1.02 | 1.09 | 0.00472 |
| Gender | 0.886 | 0.397 | 1.98 | 0.76696 |
| N | 4.86 | 1.16 | 20.2605 | 0.03005 |
| M | 4.2 | 1.68 | 10.4976 | 0.0021 |
| Grade | 1.76 | 0.6234 | 4.9694 | 0.28574 |
| Glycolytic | 0.727 | 0.3021 | 1.7482 | 0.47601 |
| Cholesterogenic | 0.319 | 0.126 | 0.8121 | 0.01654 |

| PD-L1^low^ | THCA(OS) | | | |
| --- | --- | --- | --- | --- |
| Variable | Multivariate (HR, 95% CI) | | | P-value |
| Age | 1.26 | 0.98 | 1.62 | 0.0752 |
| Gender | 2.661E-10 | 0 | Infinite | 0.9993 |
| T | 3.07 | 0.00 | 4.60E+07 | 0.8942 |
| N | 3.19 | 0.17 | 60.90 | 0.4415 |
| M | 0.10 | 0.00 | 2.27 | 0.1462 |
| Stage | 1.64 | 0.00 | 2.14E+07 | 0.9531 |
| Glycolytic | 2.09E+10 | 0.00 | Infinite | 0.9995 |
| Cholesterogenic | 3.83E+09 | 0.00 | Infinite | 0.9996 |

| PD-L1^high^ | BLCA(OS) | | | |
| --- | --- | --- | --- | --- |
| Variable | Multivariate (HR, 95% CI) | | | P-value |
| Age | 1.0152 | 0.9802 | 1.051 | 0.3989 |
| Gender | 0.8578 | 0.401 | 1.835 | 0.6925 |
| T | 0.91 | 0.4071 | 2.034 | 0.8182 |
| N | 2.507 | 1.0928 | 5.751 | 0.0301 |
| M | 1.3289 | 0.6642 | 2.659 | 0.4217 |
| Stage | 2.1339 | 0.5119 | 8.896 | 0.2981 |
| Glycolytic | 0.4288 | 0.1263 | 1.456 | 0.1746 |
| Cholesterogenic | 1.4899 | 0.6238 | 3.559 | 0.3694 |

| *Abbrev:* HR, hazard ratio; CI, confidence interval. | |
| --- | --- |
| Significant *p* < 0.05 is highlighted red. |  |
