# Supplementary figures and images for: Integrated mPD‐L1 and metabolic analysis identifies new prognostic subgroups in lung cancers with wild‐type EGFR
Source: Clin Transl Med. 2021 Dec 19;11(12):e612. doi: 10.1002/ctm2.612 (PMC8684767; doi:10.1002/ctm2.612)

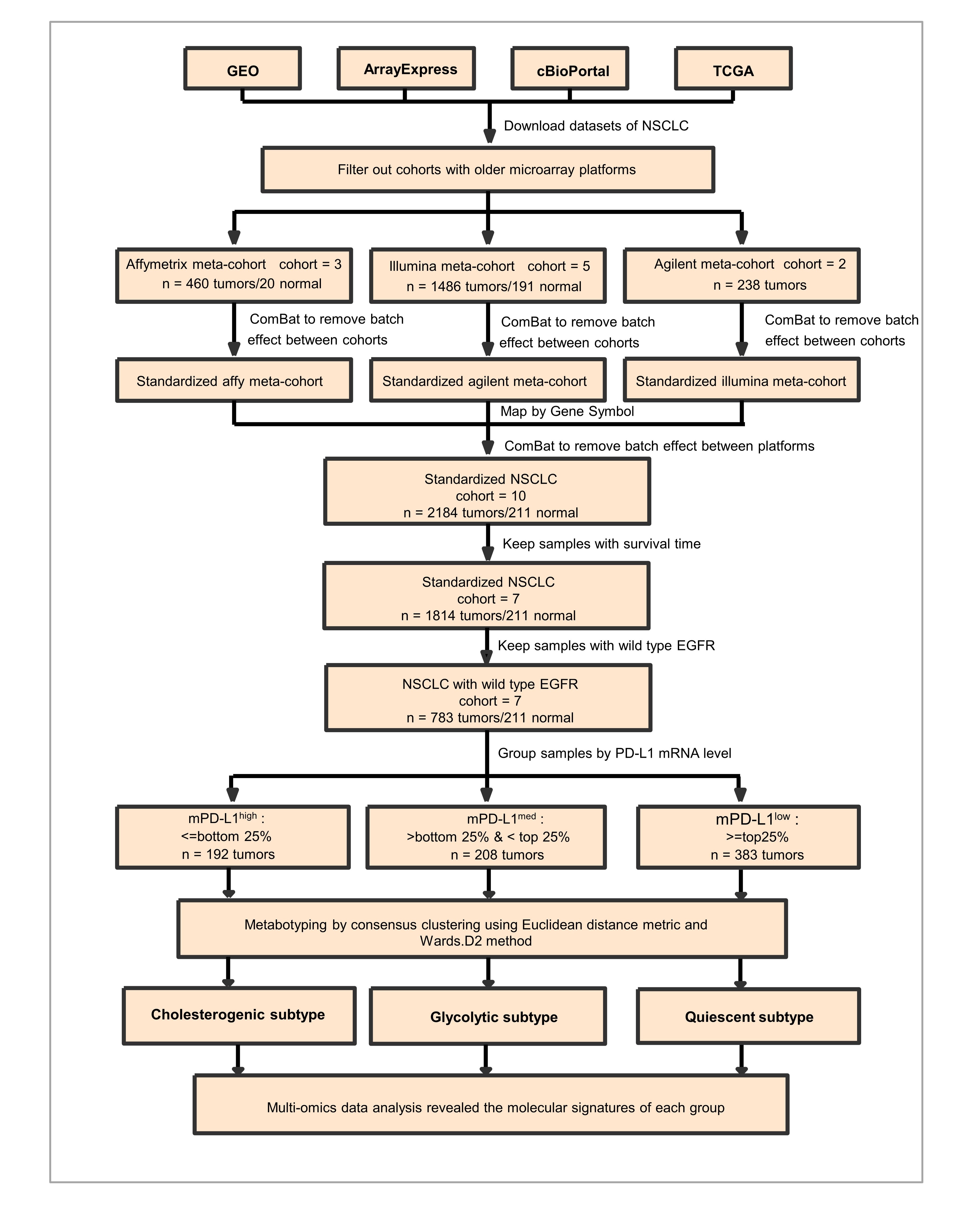

Supplement: Supplementary file 2 — SUPPORTING INFORMATION [file CTM2-11-e612-s002.jpg]

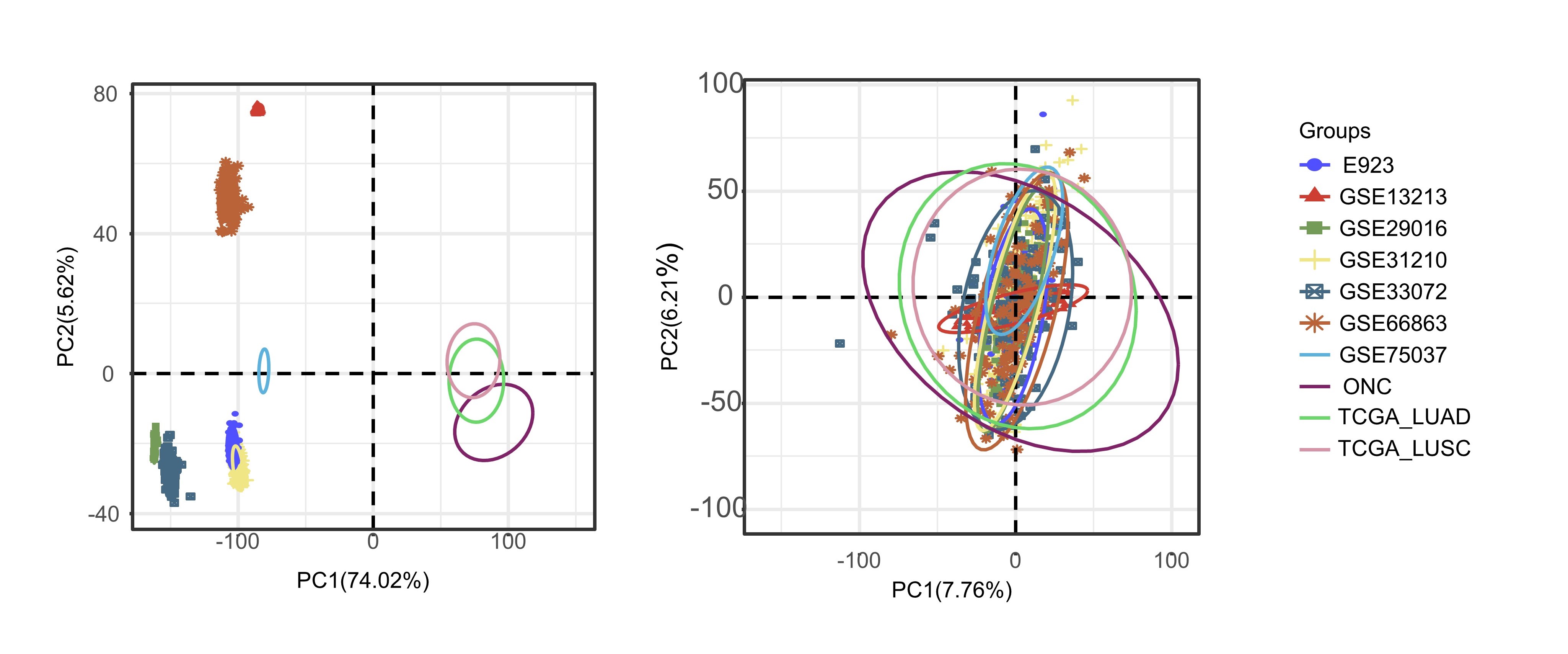

Supplement: Supplementary file 3 — SUPPORTING INFORMATION [file CTM2-11-e612-s004.jpg]

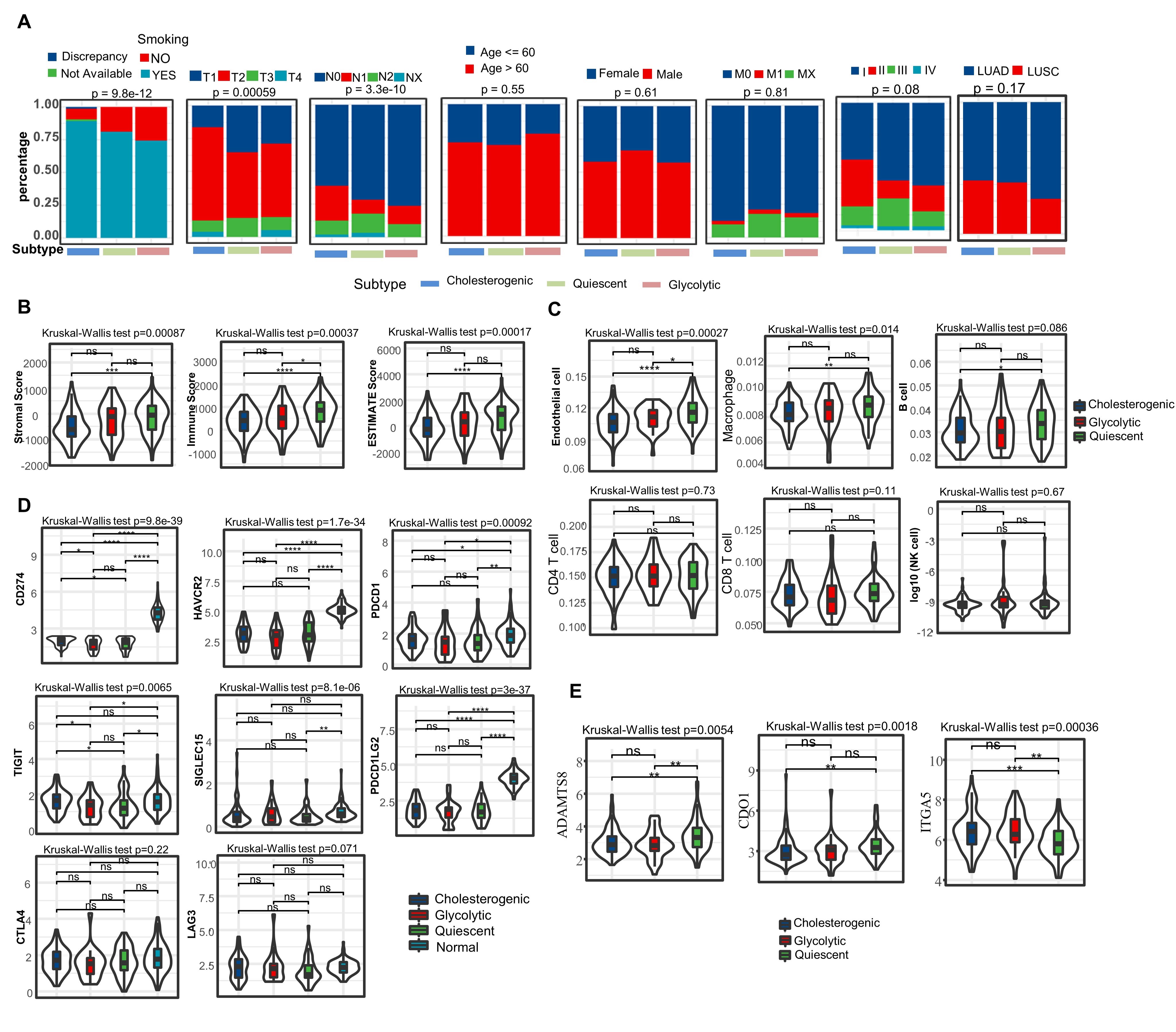

Supplement: Supplementary file 4 — SUPPORTING INFORMATION [file CTM2-11-e612-s003.jpg]

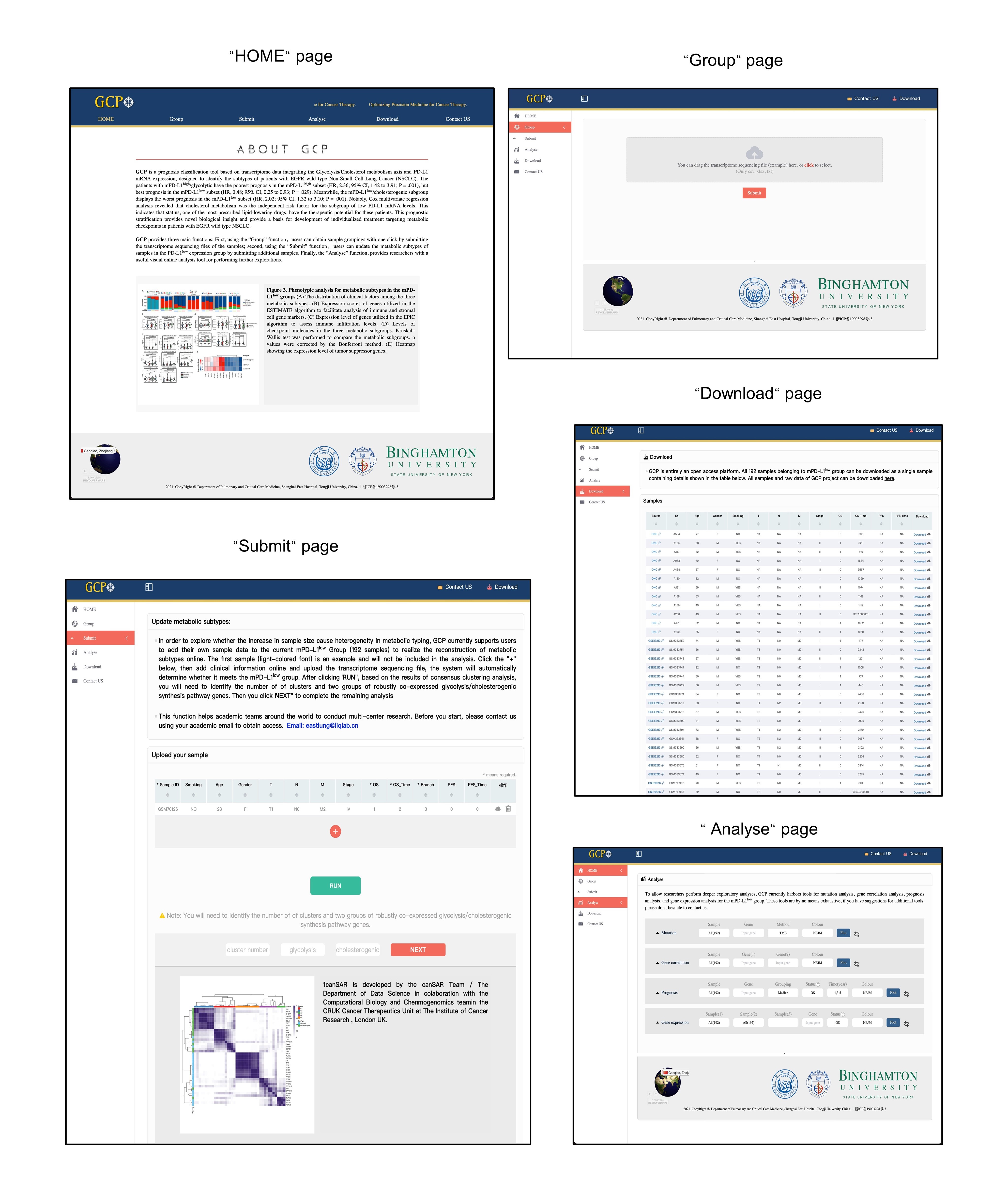

Supplement: Supplementary file 5 — SUPPORTING INFORMATION [file CTM2-11-e612-s005.jpg]
